# Progress in Medicine

**Published:** 1895-07-27

**Authors:** 


### Progress in Medicine.

### DISEASE OF THE RESPIRATORY SYSTEM.

(*Continued from page* 271.)

**Treatment of Phthisis.** — The creosote treatment was placed on a firm foundation by the experiments last summer of W. K. Fyffe, who showed that in patients taking thirty-six minims daily the virulence of the bacilli was very greatly diminished, so that it seems clear that the drug acts not only by improving digestion, but also by weakening or destroying the microbes.[1] Eck recommends it in the treatment of scrofulous children, and notices, by the way, that it causes the disappearance of freckles even in healthy persons.[2] Elvy uses a mixture of creosote, iodoform, and salol in olive oil for rectal injections.[3] The value of rectal administration, especially for local infection, is attested by W. Bodenhamer after an experience of fifty years.[4] A case of poisoning from guiacol has been carefully described by Oscar Wyss. A girl of nine years took by mistake 1¼ drachms, she became cyanotic; vomiting, apathy, dark-coloured bile, stained urine, and rapid weak pulse were followed by death.[5] Hölscher has now treated 160 cases with guiacol carbonate, which he thinks preferable to creosote, and which causes no digestive disturbance, but he regards it as useless in acute tuberculosis.[6] Bartozeuricz, and a writer in the "Journal des Practiciens," on the other hand, have found external applications of eight to thirty grains of guiacol of great and lasting value in this disease, the fever disappearing and the nutrition improving.[7] Any bad symptoms are due to impurities in the drug. Bosc also related a case of very rapid improvement in acute pulmonary tuberculosis where guiacol was painted on the backs of the hands. The results were marked and lasting.[8] Bugnion found that in rabbits external applications had no effect on acute miliary tuberculosis, but he suggested that the good results reported in human beings may be due to oxidation of the toxalbumins and other causes.[9] The chlorophenols have been found most efficacious by Simanowsky in the treatment of laryngeal tuberculosis as local applications.[10] Scarpa, having used ichthyol in 150 cases of phthisis, prefers it even to guiacol for the value of its results. He gave daily from 20 to 200 drops of a one in three solution made from a pure sample of the drug, and was able to confirm the favourable report which Cohn recently made of it.[11] Injections of a 1 per cent. solution of iodine subcutaneously have been recently employed for lung, glandular, and joint diseases by Durante and Merietta and others with some success,[12] but Vitvitsky has pointed out that in some cases of pulmonary tuberculosis iodine salts are positively harmful, causing congestion, softening, and rapid spread of the disease. The instance he adduces, however, seems capable of various interpretations.[13]

The opposite action of ergotine in restraining congestion accounts for the remarkable improvement often seen when it is administered in phthisis according to the views of T. Crocq.[14] Papers on the use of gymnastic exercises to increase apical expansion and chest capacity have appeared, recommending this method in the incipient stage. W. H. Weaver advises that efforts at forced respiration, with compression of the lower ribs while the breath is held, should be made carefully many times a day,[15] while Butler uses a series of passive and active movements, with massage, for the same purpose.[16] Further investigations on the value of peptomangan in the anæmia of phthisis have been made by K. von Ruck, showing its palatableness, the rapid gain in hæmoglobin and red corpuscles, and the degeneration of the bacilli during its use, and confirming the reports of previous observers.[17] Sacaze speaks highly of the effect of chloralose in overcoming insomnia and night sweats in all but a few cases. He gives a dose of three-quarters of a grain, repeated, if necessary, at intervals of half an hour till four doses have been taken. Quinine may be added if there is hyperrexia.[18] Conklin had best results in night sweats from agaricin gr. $\frac{1}{12}$, repeated after five hours if necessary. It can be used for any length of time without bad effects.[19] Victor Vaughan has formulated the results of his yeast nuclein treatment. He found that animals can be made for a time immune against pneumonia by injections of yeast nuclein, and that some immunity and arrest of tuberculosis can be obtained in the same way from the stimulus the nuclein gives to the protective forces of the body. In early cases of phthisis in human beings and in urinary tuberculosis great results have been obtained, but where there are cavities it is quite useless, though it may retard the progress of chronic cases. Indiscriminate and excessive use of nuclein is harmful.[20] Much interest has been excited in America by this treatment. Petteruti announces that two cases treated three years ago by cantharidinate of potash have remained completely cured, and a third practically so.[21] G. W. Balfour reports similarly of the effects of tuberculin, but his cases do not seem to be very satisfactory.[22] Klebs, however, has at last produced a purified tuberculin "antiphthisin," with which he has obtained the complete cure of tuberculosis in guinea pigs, and good results in from 75 to 90 per cent. in human beings at all stages. At the Winyah Sanitorium K. von Ruck has treated nearly sixty patients with it, and reports astonishing results even in a case of acute miliary tuberculosis. Marked improvement took place in all but five persons. In three hopeless cases, which died, the post-mortem examination showed that no recent lesions were present, and healing of much of the tissue had taken place. He noticed that the fever, physical signs, sputa, and bacilli were rapidly and steadily affected in the patients treated, and tubercular ulcers were cured quickly by local applications. Altogether, the results of antiphthisin far exceeded those obtained previously in the institution by any other method.[23]

Klebs in his recent monumental work on the subject claims that a complete reorganisation of tuberculous tissue may take place and that destruction of the bacilli may be brought about by it without injury to the tissues of the body, a claim which von Ruck's results appear to substantiate to a great extent.[24] Paquin has employed the serum of immunised horses in over twenty cases with favourable results. No reaction followed the injection of the serum, which takes some months to prepare. Richet, too, found that guinea pigs inoculated[25] with microbial serum resisted for a long time and sometimes recovered from avian tuberculosis, while the control animals died quickly.[26] Intra-laryngeal injections in the treatment of phthisis, gangrene, bronchiectasis, hæmoptysis, &c., have been largely used by several authorities. Rosenberg's solution (containing guiacol two parts, menthol 10 parts, and oil 88), solutions of menthol in Price's glycerine, or of turpentine (for hæmorrhage) have been advocated by Colin Campbell, J. K. Fowler, and Mitchell Bruce. One to two drachms are injected quickly through the glottis during inspiration, the patient being turned on the affected side after the injection is made.[27]

[1] Lanc., Sep. 22, 1894. [2] A. M. S. Bulletin, Dec. 15. [3] Am. J. Med. Sc., Nov. [4] N. Y. Med. Jour., Oct. 20. [5] Am. J. Med. Sc., Dec. 5. [6] B. M. J., Jan. 12. [7] N. Y. Med. Jour., Feb. 2. [8] B. M. J., Dec. 15. [9] B. M. J., Ap. 27. [10] Les. D. Remedes, Oct. 24. [11] B. M. J., Mar. 30. [12] Lanc., Feb. [13] Med. Week, Oct. 26. [14] Am. J. Med. Sc., Nov. [15] N. Y. Med. Times, xij., 1894. [16] Med. Jour., Oct. 20. [17] N. Y. Med. Jour., Dec. 15. [18] N. Y. Med. Jour., Dec. 8. [19] Chir. Week, Mar. 6. [20] B. M. J., Feb. 3, and Ther. Gazette, Ap. 15. [21] Am. J. Med. Sc., Jan., 1895. [22] Ed. Med. Jour., May. [23] Ther. Gazette, Mar. [24] Du Causull Behund lenz. Der Tubeveselose. [25] B. M. J., Ap. 20. [26] Med. Week, Jan. 8. [27] B. M. J., Dec. 1.

